# Identification of single nucleotide polymorphism in protein phosphatase 1 regulatory subunit 11 gene in Murrah bulls

**DOI:** 10.14202/vetworld.2017.244-248

**Published:** 2017-02-23

**Authors:** Varsha Jain, Brijesh Patel, Farhat Paul Umar, H. M. Ajithakumar, Suraj K. Gurjar, I. D. Gupta, Archana Verma

**Affiliations:** 1 Division of Animal Genetics and Breeding, ICAR-National Dairy Research Institute, Karnal, Haryana, India; 2 Livestock Production Management Section, ICAR-National Dairy Research Institute, Karnal, Haryana, India; 3 Division of Animal Physiology, ICAR-National Dairy Research Institute, Karnal, Haryana, India

**Keywords:** Murrah bulls, protein phosphatase 1 regulatory subunit 11, polymerase chain reaction, restriction fragment length polymorphism

## Abstract

**Aim:**

This study was conducted with the objective to identify single nucleotide polymorphism (SNP) in protein phosphatase 1 regulatory subunit 11 (PPP1R11) gene in Murrah bulls.

**Materials and Methods:**

Genomic DNA was isolated by phenol–chloroform extraction method from the frozen semen samples of 65 Murrah bulls maintained at Artificial Breeding Research Centre, ICAR-National Dairy Research Institute, Karnal. The quality and concentration of DNA was checked by spectrophotometer reading and agarose gel electrophoresis. The target region of PPP1R11 gene was amplified using four sets of primer designed based on *Bos taurus* reference sequence. The amplified products were sequenced and aligned using Clustal Omega for identification of SNPs. Animals were genotyped by polymerase chain reaction-restriction fragment length polymorphism (PCR-RFLP) using EcoNI restriction enzyme.

**Results:**

The sequences in the NCBI accession number NW_005785016.1 for *Bubalus bubalis* were compared and aligned with the edited sequences of Murrah bulls with Clustal Omega software. A total of 10 SNPs were found, out of which 1 at 5’UTR, 3 at intron 1, and 6 at intron 2 region. PCR-RFLP using restriction enzyme EcoNI revealed only AA genotype indicating monomorphism in PPP1R11 gene of all Murrah animals included in the study.

**Conclusion:**

A total of 10 SNPs were found. PCR-RFLP revealed only AA genotype indicating monomorphism in PPP1R11 gene of all Murrah animals included in the study, due to which association analysis with conception rate was not feasible.

## Introduction

The livestock sector is an integral component of agricultural production system and tool for socioeconomic development and it contributes 4.11% of total gross domestic product (GDP) and 27.25% of agricultural GDP [1]. India possesses the largest buffalo population in the world with high genetic diversity consisting of 13 recognized breeds. Buffalo population in India is 108.7 million that consists only 21.23% of total livestock population. At present, buffalo produce 67.67 MT of milk contributing 51.1% of the total milk [2]. Country has achieved the highest milk production of 154.9 MT in the year 2015-16, which has led to increase in the per capita availability of milk up 337 g/day by 2015-16 (www.nddb.org).

Murrah buffalo is one of the most important and well-known water buffalo breed in the world with high milk production. Its home tract lies in Rohtak, Hisar, Jind, and Gurgaon districts of Haryana. Murrah buffaloes produce around 2000 kg of milk. The Murrah breed is being used for upgrading local buffaloes in many parts of Asia and other parts of the world. Due to the availability of frozen semen, the demand for the elite buffalo males is increasing day by day.

Bull fertility, an economically important complex trait, is controlled by genetic as well as environmental factors. Several studies conducted in different species highlighted the role of different genes during the process of male reproduction and the cascade of fertilization. However, the reports on genetic control of fertility in bulls are scanty and need extensive investigation to meet the future needs. It is thus important to develop new molecular tools to accurately estimate fertility levels. Genetic markers can be useful for selection of breeding bulls and ensuing improvement of buffalo population.

Protein phosphatase 1 regulatory subunit 11 (PPP1R11) gene is one of the important fertility-related genes which was found to be associated with the sire conception rate (SCR) [3,4]. This gene encodes a specific inhibitor of PP1 with a differential sensitivity toward the metal-independent and metal-dependent forms of PP1.

## Materials and Methods

### Ethical approval

The experimental plan of the study was duly approved by the Institution Animal Ethics Committee of National Dairy Research Institute (NDRI), Karnal, Haryana, India.

### Resource population

Frozen semen samples from a total of 65 Murrah bulls maintained at Artificial Breeding Research Centre (ABRC), ICAR-NDRI, Karnal, were included in the study.

### Genomic DNA extraction

Phenol-chloroform method as described by Sambrook and Russell [5] with minor modifications was used for DNA isolation from semen of Murrah bulls. The quality of DNA was checked by 1.5% agarose gel electrophoresis. Quality and quantity of DNA was estimated by Nanodrop spectrophotometer (ND 1000). Optical density (OD) was determined at wavelengths 260 nm and 280 nm in ultraviolet (UV)-visible spectrophotometer against distilled water as blank sample. DNA samples with OD_260_/OD_280_ ranging between 1.7 and 1.9 were of good quality. The genomic DNA was diluted to a final concentration of 30 ng/µl and stored at −20°C.

### Primer designing

Four sets of forward and reverse gene-specific oligonucleotide primers based on reference (AC_000180.1) gene sequence available at NCBI database (http://www.ncbi.nlm.nih.gov) were designed to amplify bovine PPP1R11 gene using Primer 3 software (http://www.primer3.ut.ee). The specificity of the designed primers was checked by BLAST program. Primers were procured from Eurofins Genomics India Pvt. Ltd. (Bengaluru). The sequence of primers, their nucleotide numbers, target region, and amplicon sizes is given in Table-1.

**Table-1:** Sequence of the primers designed for amplification of targeted region of PPP1R11 gene in Murrah bulls.

Primer code	Sequence (5’-3’)		Targeted region	Amplicon size (bp)	T_a_ (°C)
P1	F	AGCGCTTTGACGCATTTAGT	5’flanking, exon 1	599	55.6
	R	GCCAAGTTCCCAGTCTTTCA			
P2	F	TGGGATGGTGGTGTCTGTAA	Intron 1	596	56.5
	R	CCCAGCAAATCTTCAAAGGA			
P3	F	ATTTGGGGAGGAAGATGGAG	Partial intron 1, exon 2	441	56.5
	R	TGTGGGGCAACAATTACTCA			
P4	F	ACCATCAAACTTCGGAAACG	Partial exon 2, intron 2	472	58.2
	R	TTCCATTCCCACTGGATCTC			

PPP1R11=Protein phosphatase 1 regulatory subunit 11

### Polymerase chain reaction (PCR) amplification and DNA sequencing

**Figure-1:**
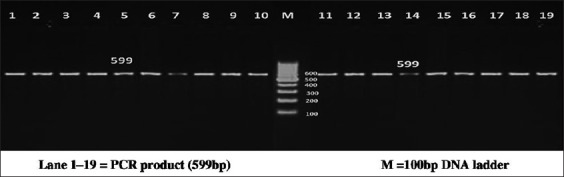
Resolution of polymerase chain reaction (PCR) amplified product of Primer 1 on 2% agarose gel. Lane 1-19=PCR product (599 bp), M=100 bp DNA ladder.

**Figure-2:**
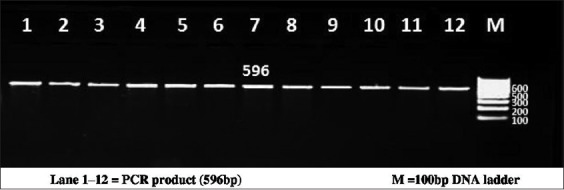
Resolution of polymerase chain reaction (PCR) amplified product of Primer 1 on 2% agarose gel. Lane 1-12=PCR product (596 bp), M=100 bp DNA ladder.

**Figure-3:**
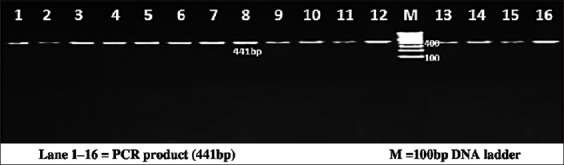
Resolution of polymerase chain reaction (PCR) amplified product of Primer 3 on 2% agarose gel. Lane 1-16=PCR product (441 bp), M=100 bp DNA ladder.

**Figure-4:**
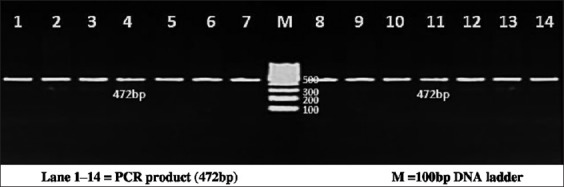
Resolution of polymerase chain reaction (PCR) amplified product of Primer 4 on 2% agarose gel. Lane 1-14=PCR product (472 bp), M=100 bp DNA ladder.

The PCR reactions were carried out on a total of 25 µl volume containing template DNA of 3 µl (30 ng/µl), 0.5 µl of forward and reverse primer, PCR Master Mix (×2) (Fermentas) of 12.5 µl, and 9.5 µl of nuclease free water. Each 0.2 ml tube containing PCR reaction cocktail was kept in thermal cycler (BioRad, T100) for amplification of region of bovine PPP1R11 gene. PCR conditions were optimized at an initial denaturation at 95°C for 3 min, followed by 35 cycles with initial denaturation at 94°C for 30 s, annealing temperature of 55.6°C, 56.5°C, 56.5°C and 58.2°C, respectively, for each primer set for 30 s, extension at 72°C for 1 min followed by a final extension at 72°C for 5 min. PCR products were detected by electrophoresis on 2% agarose gel stained with ethidium bromide using 100 bp DNA ladder (Figures-1-4).

The representative samples of amplified PCR products from four set of primers were sent to 1^st^ base sequencing INT (Malaysia) for purification and custom sequencing from both ends (5’ and 3’ ends). DNA sequencing results for the respective region of bovine PPP1R11 gene were visualized and edited using BioEdit software. Each edited sequence was aligned with corresponding reference sequence using Clustal Omega multiple sequence alignment program for DNA (www.ebi.ac.uk/Tools/msa/clustalo) to identify single nucleotide polymorphisms (SNPs).

### PCR-restriction fragment length polymorphism (PCR-RFLP) analysis

**Figure-5:**
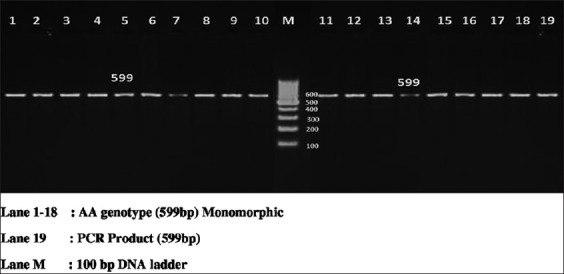
Polymerase chain reaction (PCR)-restriction fragment length polymorphism of primer 1 of protein phosphatase 1 regulatory subunit 11 gene using EcoNI restriction enzyme in Murrah animals. Lane 1-18=AA genotype (599 bp) monomorphic, Lane 19: PCR product (599 bp), Lane M: 100 bp DNA ladder.

Preliminary selection of the restriction enzyme to be used was done using NEBcutterV2.0 by submitting *Bubalus bubalis* reference and alternate sequence derived from the results of sequencing of primer 1 (5’UTR and exon1), primer 2 (intron 1), primer 3 (partial intron 1, exon 2), and primer 4 (partial exon 2, intron 2). *Eco*NI was the only enzyme which produces restriction fragments at primer 1 region. Thus, identified *Eco*NI was used to genotype the targeted SNPs in remaining samples of Murrah bulls. The PCR products (10 µl) of each animal were digested with *Eco*NI restriction enzyme; reaction mixture was centrifuged for few seconds for uniform mixing and then incubated at 37°C for 12-16 h. Restriction fragments were resolved on 2.5% agarose gel electrophoresis and visualized by ethidium bromide staining. The restriction digested gene fragment were visualized on UV transilluminator and photographed with gel documentation system (Figure-5).

### Estimation of conception rate

Data were recorded for the bulls, and their mates from the records maintained at ABRC, Animal Genetics and Breeding Division, and Livestock Research Centre, ICAR-NDRI, Karnal, Haryana.

The conception rate of bulls was calculated according to the given formula:


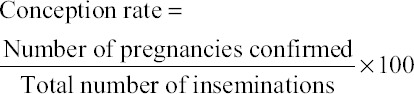


## Results

For determining the SNPs in PPP1R11 gene in Murrah bulls, the sequences in the NCBI accession number NW_005785016.1 for *B. bubalis* were compared and aligned with the edited sequences of Murrah bulls with Clustal Omega software. A total of 10 SNPs were found, out of which 1 at 5’UTR, 3 at intron 1 and 6 at intron 2 (Table-2). The PCR product of primer1 (599 bp) was digested with *Eco*NI restriction enzyme. The PCR-RFLP products using restriction enzyme *Eco*NI revealed monomorphism with AA genotype in PPP1R11 gene of all Murrah animals included in the study (Table-3).

**Table-2:** SNPs in PPP1R11 gene in Murrah buffalo.

Nucleotide position	*B. bubalis* (NW_005785016.1)	Murrah (present study)	Region
85	T	A	5’UTR
524	G	C	Intron1
536	C	A	
886	G	A	
1526	C	T	Intron2
1850	G	T	
1856	C	A	
1863	T	C	
1873	C	A	
1878	T	G	

*B. bubalis*=*Bubalus bubalis*, PPP1R11=Protein phosphatase 1 regulatory subunit 11, SNP=Single nucleotide polymorphism

**Table-3:** Restriction fragment sizes and corresponding genotypes of PPP1R11 gene in Murrah buffaloes using primer restriction enzyme combination.

PCR products	Enzyme	Number of cutting sites	Fragments (bp)	Result	Genotype
Primer 1	*Eco*NI	0	599	Monomorphic	AA

PPP1R11=Protein phosphatase 1 regulatory subunit 11, PCR=Polymerase chain reaction

Overall conception rate was estimated by total number of pregnancy in each female out of total numbers of AI and it was expressed in percentage. The overall conception rate was 39.51% which ranged from 17.86% to 62.50%. Mir *et al*. [6] estimated the average overall conception rate of Murrah bulls of NDRI herd as 39.19±1.55%.

## Discussion

Our results showed that a total of 10 SNPs were found, out of which 1 at 5’UTR, 3 at intron 1, 6 at intron 2. There is no any report on sequence characterization and SNPs in PPP1R11 gene in Murrah buffalo to compare and contrast with the findings of present study. However, results of this study are compared with the available sporadic reports in cattle. Li *et al*. [7] analyze the association between highly conserved spermatogenesis genes and SCR in US Holsteins as a measure of bull fertility. Four SNPs were reported in PPP1R11 gene in which 3 were located in intronic region while 1 SNP located in 5’UTR (untranslated region). Khatib [4] found that an SNP (rs109300808 T>G) located in 5’UTR region at position 28710268 of PPP1R11 showed significant association with SCR in the Genex population, with allele G associated with increased SCR, having an allele substitution effect of 0.15 and P-value of 0.046. Association analysis was not feasible because of monomorphic RFLP band patterns using *Eco*NI restriction enzyme for all the samples of Murrah animals. Although this study targeted only PPP1R11 gene, a few other candidate genes for bull fertility (SRY, SPP, and CRISP), explored for genetic polymorphism in our laboratory, have also exhibited monomorphism in Murrah buffalo, which may be due to allele fixation in the highly selected population.

## Conclusion

A total of 10 SNPs were found, out of which 1 at 5’UTR, 3 at intron 1 and 6 at intron 2. PCR-RFLP of using restriction enzyme *Eco*NI revealed monomorphism in PPP1R11 gene of all Murrah animals included in the study. Overall conception rate of Murrah bulls was 39.51%; however, association analysis was not feasible because of monomorphism.

## Authors’ Contributions

AV and IDG designed the research project, VJ performed wet lab work and analysis, BP helped in collection of blood samples and manuscript preparation. FPU, HMA and SKG helped in the lab work and processing of samples. All authors have read and approved the final manuscript.
